# The Impact of Disease Registries on Advancing Knowledge and Understanding of Dementia Globally

**DOI:** 10.3389/fnagi.2022.774005

**Published:** 2022-02-07

**Authors:** Shimaa A. Heikal, Mohamed Salama, Yuliya Richard, Ahmed A. Moustafa, Brian Lawlor

**Affiliations:** ^1^Institute of Global Health and Human Ecology (IGHHE), The American University in Cairo (AUC), New Cairo, Egypt; ^2^Medical Experimental Research Center (MERC), Faculty of Medicine, Mansoura University, Mansoura, Egypt; ^3^Blue Horizon Counseling Services, Sydney, NSW, Australia; ^4^School of Psychology, Faculty of Society and Design, Bond University, Gold Coast, QLD, Australia; ^5^Department of Human Anatomy and Physiology, The Faculty of Health Sciences, University of Johannesburg, Johannesburg, South Africa; ^6^Trinity College Institute of Neuroscience, Trinity College Dublin, Dublin, Ireland

**Keywords:** dementia, Alzheimer’s disease (AD), dementia datasets, disease registries, dementia prevalence, dementia treatment

## Abstract

To help address the increasing challenges related to the provision of dementia care, dementia registries have emerged around the world as important tools to gain insights and a better understanding of the disease process. Dementia registries provide a valuable source of standardized data collected from a large number of patients. This review explores the published research relating to different dementia registries around the world and discusses how these registries have improved our knowledge and understanding of the incidence, prevalence, risk factors, mortality, diagnosis, and management of dementia. A number of the best-known dementia registries with high research output including SveDem, NACC, ReDeGi, CREDOS and PRODEM were selected to study the publication output based on their data, investigate the key findings of these registry-based studies. Registries data contributed to understanding many aspects of the disease including disease prevalence in specific areas, patient characteristics and how they differ in populations, mortality risks, as well as the disease risk factors. Registries data impacted the quality of patients’ lives through determining the best treatment strategy for a patient based on previous patient outcomes. In conclusion, registries have significantly advanced scientific knowledge and understanding of dementia and impacted policy, clinical practice care delivery.

## Introduction

Dementia, Alzheimer’s disease (AD) and other neurodegenerative diseases are considered to be a major societal challenge (Weiner et al., 2018). The advancements in education, housing and sanitation, as well as, improvements in healthcare services, have all contributed to increasing people’s life span and decreasing mortality. However, living longer has resulted in an increase in the prevalence of age-related conditions, including dementia. Dementia is a devastating disorder that is defined as “A syndrome, usually of chronic or progressive nature, caused by a variety of brain illnesses that affect memory, thinking, behavior and ability to perform everyday activities” (WHO, 2012). The number of people with dementia is estimated to triple in 2050, and hence, the disease is considered a public health priority by the WHO (2018). In addition to the impairment in memory and cognition, dementia affects day to day function and leads to an increased dependency (Liu-Seifert et al., 2015). The estimated annual cost of dementia care per person is estimated to be $32865 in high income countries, $6827 in upper-middle income countries and $3109 in lower middle-income countries (WHO, 2017). A comprehensive approach to patient care that results in an improvement in patients’ lives will be an effective way to decrease the costs and address this global health burden. The availability of accurate disease data is crucial to determine the risk factors of the disease and identify targets for treatment and prevention.

Disease registries are considered one of the most cost-effective ways of collecting patient information and longitudinal follow-up data that can be used for research purposes as well as clinical observations (Gliklich et al., 2012). The availability of datasets together with the advances in data analytic techniques has allowed the reporting of insightful new data and perspectives in the field of dementia. This paper aims to review studies that were based on registry data and evaluate the impact of these studies on advancing the diagnostic and treatment procedures for better disease monitoring, as well as, increasing our knowledge in the field. Specifically, this review (1) describes the publication output of a number of the best-known dementia registries with high publication output, particularly SveDem, NACC, ReDeGi, CREDOS and PRODEM, (2) investigates the objectives and key findings of the registry-based studies to discuss how we have benefited from the data in terms of knowledge of the incidence of dementia in different populations, as well as the factors that increase patients’ mortality risk, and (3) studies the impact of registry findings on disease management and improved diagnostic procedures, treatment practices and advance our general knowledge of the disease will also be discussed.

## Background: Dementia Registries Around the World and Their Importance

The imperative to tackle the impact of chronic diseases has led to the establishment of disease registries to collect patient data and gain insights into the cause and pathogenesis of these illnesses (Richesson and Vehik, 2010). Registries differ in definitions and classifications according to their purpose, reporting method and the type of data included (Richesson and Vehik, 2010); however, a registry is generally a system of ongoing documentation of patients’ data with a particular disease (Krysinska et al., 2017). Different types of patient registries are increasingly being developed to address the needs of healthcare systems all over the world (Hopper, 2017).

The first disease registries were developed in Scandinavian countries such as Denmark, Finland and Sweden and have continued to grow to include many different diseases and data from medical records including demographic and personal characteristics (Frank, 2000). In contrast, to our knowledge, the United States was the first country to establish a registry for dementia disorders when they created the Consortium to Establish a Registry for Alzheimer’s Disease (CERAD) that aimed to evaluate the accuracy of AD diagnosis (Krysinska et al., 2017). Subsequently, other dementia registries were developed all over the world consisting of large databases of dementia patients to enable studies on causes, risk factors, prevalence and other variables related to the disease, e.g., London, Newcastle, and the Creutzfeldt-Jakob register in Edinburgh (Leach and Levy, 1993). The National Alzheimer’s Coordinating Center (NACC) is one of the older registries that was established in the United States in 1990 to collect standardized clinical data on patients with Alzheimer’s disease (Beekly et al., 2004). The NACC includes standardized and longitudinal data in addition to the autopsy data for patients with AD that have been used in studies to investigate symptoms, diagnosis, genetics, biomarkers, as well as clinical practices and mortality risk in dementia (Krysinska et al., 2017).

More recently, dementia research registries have been developed in Asia and Europe. The Clinical Research Center for Dementia of South Korea Study (CREDOS) initiated a longitudinal dementia registry in 2005 with a focus on specific dementia subtypes including AD to collect data on demographic and patient characteristics and to assess disease prevalence (Park et al., 2011; Hye Choi et al., 2012; Kim et al., 2014)The Prospective Dementia Registry Austria (PRODEM) was established in 2008 as a longitudinal cohort of AD patients to collect data on clinical evaluation, magnetic resonance (MR) imaging and diagnostic markers (Seiler et al., 2012). Some registries have used an epidemiological surveillance model where specific populations are maintained under surveillance to provide standardized data. For example, the Registry of Dementias of Girona (ReDeGi) was set up in 2007 in Spain to record all new cases of dementia in the region of Girona and provide information about the demographic and clinical characteristics of dementia patients in the region (Garre-Olmo et al., 2009). The quality-of-care registries are among the most successful registry types (Hopper, 2017; Krysinska et al., 2017). The Swedish Dementia Registry (SveDem) is a national, online dementia registry that was established in 2007 to evaluate the quality of patient care and the adherence to national guidelines (Fereshtehnejad et al., 2015; Religa et al., 2015). SveDem includes more than 74,000 registered patients as of 2017 and collects data about diagnostic work-up and treatment. Quality of care registries are extremely beneficial as they focus on improving patients’ quality of lives which is the reason of the current shift toward creating this type of registries. The availability of common patients’ data that are usually collected in these types of quality-of-care registries makes it possible to link the registry with other patient or disease registries in the region. Using multiple data sources for a patient is tremendously valuable in finding the association between the disease and other comorbidities or risk factors. SveDem, for example, was merged with the Swedish Heart Failure Registry (RiksSvikt) (Cermakova et al., 2015), as well as the Swedish stroke registry (Riksstroke) (Zupanic et al., 2020). Many studies have analyzed data from these registries together to provide more insights concluded from these large sets of data.

The feasibility of creating new dementia registries is being explored in several countries. Hopper et al., assessed the feasibility of creating a national dementia registry for Ireland, discussed the benefits, anticipated outcome, most suitable strategies for constructing and managing the registry, and the most effective registry structure needed (Hopper, 2017). Most recently, a multi-partner project was initiated by the Latin American and Caribbean Consortium on Dementia (LAC-CD) to expand dementia research in the participating regions. The consortium’s objective is to collect genetic, neuroimaging and behavioral data to improve dementia diagnosis and help developing new interventions that benefit the diverse populations (Projects – LAC-CD, 2020).

Although many registries were already established and published studies based on their data, yet many of them still face some limitations. One of the main restraints in any registry is the minimal set of data that can be included. For example, quality of care registries focusses on patients’ history and treatment while lacking many diagnostic data including imaging and neurological tests which might hinders some areas of research. However, prioritizing the most essential set of data to be included in the registry while excluding the less important points is a major step in the planning of a registry which guarantees its success. Creating a registry that include everything about a patient is extremely difficult considering the availability of funds, as well as the sustainability of registries (Newton and Garner, 2002). Hence, a registry board should be cautious regarding which data should be included to be benefit the registry and achieve its objectives.

## Literature Search and Data Collection

We performed a rapid literature search to identify the best-known dementia registries with high publication output to be included in the study. SveDem, NACC, ReDeGi, CREDOS, and PRODEM were selected. We then performed a comprehensive literature search to find the peer-reviewed papers that were based on the data of any of the selected registries. All the collected studies were categorized to develop common themes and sub-themes. Finally, the studies that did not fit any of the themes were filtered and we presented the data as how each of the different areas or themes benefited from the analysis of data provided by dementia registries.

## Registries Outcomes on Quantifying the Disease: Incidence, Prevalence, and Mortality

Studying the prevalence and incidence of each dementia subtype in a specific region, as well as the associated sociodemographic risk factors is crucial for effective resource utilization and disease management. In addition, comparing dementia prevalence and risk factors across regions and countries will help determine if there are specific population-related risk factors that increase the disease incidence in different countries or regions.

### Disease Incidence and Patient Characteristics

The ReDeGi registry in Spain has been facilitating studies that estimate the incidence of dementia in the population (Table 1). The validity of the registry data and the benefits of epidemiological surveillance in the specific defined geographical area that the registry covers have been reported (Garre-Olmo et al., 2009; Calvó-Perxas et al., 2012b). AD was the most frequent dementia subtype among the cases and the onset of symptoms before diagnosis was estimated to be 2.4 years. Disease risk factors included family history of dementia, history of depression and high blood pressure (Garre-Olmo et al., 2009). Another study used ReDeGi data to compare the characteristics of newly diagnosed cases of early onset dementia (EOD) and late-onset dementia (LOD) (*N* = 2083) (Garre-Olmo et al., 2010) using the traditional cutoff that define EOD as being diagnosed with an age at onset of less than 65 years old (Mendez, 2006). The incidence of dementia between the age group (50–64 years old) was significantly higher than the younger age (group 30–49 years old) and doubled with every 5-years age increase. AD was the most frequent subtype of EOD followed by secondary dementia (a form of dementia related a pre-existing mental or physical condition), vascular dementia (VaD) and then frontotemporal dementia (FTD) (Garre-Olmo et al., 2010).

**TABLE 1:** Registry-based studies on dementia incidence and patients’ characteristics.

#	Title	Year	Registry data used	Number of patients	Follow up duration	From	To	Purpose	Conclusion	References
1	Clinical and demographic characteristics of the cases of dementia diagnosed in the Health District of Girona throughout the period 2007–2010: data from the Girona Dementia Registry (ReDeGi)	2012	ReDeGi	2814		2007	2010	Investigate the frequency of the diagnoses and their clinical and sociodemographic characteristics on different subtypes of dementia	There were differences in age, sex, family history of dementia among the different dementia subtypes	Calvó-Perxas et al., 2012b
2	Incidence and subtypes of early onset dementia in a geographically defined general population	2010	ReDeGi	2083				Estimate the incidence of early onset dementia (EOD) and its compare clinical characteristics with late-onset dementia (LOD)	The incidence rate of EOD increases with increased age	Garre-Olmo et al., 2010
3	Incidence and characteristics of uncommon dementia subtypes: Results from 10 years of clinical surveillance by the Registry of Dementia of Girona	2019	ReDeGi	7357				Describe the incidence and the clinical characteristics of uncommon dementia subtypes	Age, sex, dementia severity and medical comorbidities differ with dementia subtype	Calvó-Perxas et al., 2019
4	Rate of dementia diagnoses according to the degree of aging of the population	2015	ReDeGi	4314				Analyze the rate of dementia diagnoses, dementia subtypes and patient clinical characteristics	Clinical incidence of dementia was lower in regions with high percentages of older people	Calvó-Perxas et al., 2015
5	Diagnosis of Dementia in the Specialist Setting: A Comparison Between the Swedish Dementia Registry (SveDem) and the Registry of Dementias of Girona (ReDeGi)	2016	ReDeGi/SVEDEM	22384 + 5032				Compare the frequency of dementia diagnoses from two dementia registries in Europe	There were differences in demographic characteristics, diagnostic scores, and treatment strategy among the different European cohorts	Garre-Olmo et al., 2016
6	Basic Diagnostic Work-Up Is More Complete in Rural than in Urban Areas for Patients with Dementia: Results of a Swedish Dementia Registry Study	2019	SVEDEM	58141		2007	2014	Compare the diagnostic process and the management of dementia in rural and urban areas of Sweden	The diagnostic workup differed between rural, intermediate, and urban areas.	Roheger et al., 2019
7	Dementia diagnosis differs in men and women and depends on age and dementia severity: data from SveDem, the Swedish Dementia Quality Registry	2012	SVEDEM	6937		2007	2009	Investigate the association between age and gender with dementia assessment	Elderly groups used significantly lower number of tests compared to middle-aged and young groups	Religa et al., 2012
8	Demography, diagnostics, and medication in dementia with Lewy bodies and Parkinson’s disease with dementia: data from the Swedish Dementia Quality Registry (SveDem).	2013	SVEDEM	784		2007	2011	Compare the characteristics of DLB and PDD patients		Fereshtehnejad et al., 2013
9	No Significant Difference in Cognitive Decline and Mortality between Parkinson’s Disease Dementia and Dementia with Lewy Bodies: Naturalistic Longitudinal Data from the Swedish Dementia Registry	2018	SVEDEM	1110 DLB + 764 PDD			Dec 30th 2015	Compare cognitive decline and mortality between patients with Dementia with Lewy Bodies and Parkinson’s Disease Dementia	There is no significant difference in rate of cognitive decline and mortality between DLB and PDD	Fereshtehnejad et al., 2018

Although AD and VaD are highly frequent, the clinical characteristics of the uncommon dementia subtypes were also investigated as they are considered a challenge to diagnose. Data from ReDeGi showed that these less common subtypes were more frequent in people aged over 65 years. Non-AD neurodegenerative diseases represented the highest incidence rate among patients, with dementia with Lewy body (DLB) and Parkinson’s disease dementia (PDD) being the most frequent subtypes. Demographic and clinical characteristics also differed, for example, non-AD neurodegenerative dementia and dementia due to multiple causes occurred mainly in men and those that were older. In contrast, women were predominantly represented in those with dementia associated with a mental health disorder, as a higher number of women had a history of depression or were referred from mental health service units (Calvó-Perxas et al., 2019).

Environmental factors were also found to affect the clinical manifestations of the disease. The cost and accessibility to healthcare services, as well as aging among the population were major contributors to the incidence rate of dementia. Clustering cities into aged or young regions according to the number of older people living in the city showed that the incidence rate of dementia diagnosis is lower in the aged cities. This phenomenon may be partly explained by the adaptation of the environment docility hypothesis in that the surrounding environment may influence an individual living in young region to look for help and hence get an early diagnosis. In contrast, living in aging municipalities, there is less cognitive pressure on performing daily activities (Morgan et al., 1984). As the required cognitive functions in performing everyday activities are lower, symptoms of dementia will not become evident and fewer cases of dementia are diagnosed (Calvó-Perxas et al., 2015). All in all, the ReDeGi registry provided researchers with a significant amount of data on dementia patients that allowed them to determine the incidence of dementia in the population, the most frequent subtypes, the patients’ characteristics, and the environmental factors contributing to these characteristics (Table 1).

The Swedish registry, SveDem, has had a major role in facilitating dementia epidemiological studies as well. Religa et al. (2012) examined the differences in age and gender among SveDem dementia patients (*N* = 6937) and showed that age and severity of dementia predict the number of diagnostic tests that patients can complete. Diagnostic workups were significantly higher among younger patients who were able to complete more tests and had higher MMSE scores in comparison to lower diagnostic workups in older individuals who also had lower MMSE scores. Religa et al. (2012) also reported some gender differences in workup as men were more likely to complete higher number of diagnostic tests, in comparison to females. Taken together, the adequate number of dementia diagnostic tests should be adapted based on the age and dementia severity of the participants. In other words, using a fewer number of tests will help older individuals complete the diagnostic workup, and thus provide a more accurate diagnosis (Religa et al., 2012). In contrast, two other groups of DLB and PDD patients did not show any differences except a significant lower age of disease-onset and less cognitive impairment in the PDD than in DLB group (Fereshtehnejad et al., 2013, 2018). Additionally, the diagnostic process and disease management were investigated in rural and urban areas of Sweden as the geographical variations may represent important socio-environment contributors to dementia incidence and etiology. Roheger et al. (2019) used SveDem data to demonstrate that the dementia diagnostic procedures such as the basic examination, MMSE test, blood analysis and neuroimaging were, surprisingly more complete among patients living in rural than urban or intermediate areas. They suggested that their results might be due to sampling bias or that the patients living in rural areas are more likely to be registered in SveDem and therefore have a complete diagnostic work-up. Gender was also a factor as male patients received more complete diagnosis than women did (Roheger et al., 2019). This type of study should be considered for future improvements of disease management by avoiding inequalities among rural and urban areas and ensure the inclusion of complete workup tests for all cases.

Moreover, a recent study compared the data of two dementia registries, SveDem and ReDeGi to determine the differences in subtypes frequency, demographics, and clinical manifestations. The results showed significant differences between the two cohorts in MMSE test scores and the frequency of dementia subtype among the registry participants. However, there was no significant difference in age and gender of the participants in both cohorts. SveDem cases had higher MMSE scores at diagnosis than ReDeGi cases. AD was the most frequent subtype although its percentage was higher in ReDeGi cases. On the other hand, VaD and mixed dementia accounted for higher percentages in SveDem cases. Differences in medication profiles at the time of diagnosis were also identified as patients from ReDeGi consumed higher amounts of antipsychotics than SveDem patients. The differences in results could be explained by the variability of the genetic profiles of the two populations, differences between the specialists who performed the diagnosis or by the differences in diagnostic assessment. However, additional data regarding the education level of registered cases, the severity of dementia symptoms and the length of their treatment would be helpful to fully understand the cause of these differences (Garre-Olmo et al., 2016).

The use of registry data from SveDem and ReDeGi is greatly impacting our understanding of the disease prevalence and the differences in patient characteristics through providing data from the real world (Table 1). Understanding these differences may help improve the disease prediction algorithms for more precise patient handling. However, relying on these data should be done cautiously as registries data usually have the limitation of inclusiveness. Most registries depend only on patients registered in specific hospitals or memory clinics neglecting other private or emergency geriatric centers, and more substantially the undiagnosed patients in the population. Yet, having a source of evidence that enables the estimation of epidemiological features of such an underdiagnosed disease based on real-world data is extremely necessary. These real-world data will also help the policymakers in taking their decisions of services improvement, resources and budget allocation based on real evidence.

### The Risk of Mortality

Life expectancy is severely affected by dementia. Patient survival (i.e., how many years the patient will live after diagnosis) and years of life lost after dementia diagnosis (i.e., years representing premature death) should be considered as they differ depending on the type of dementia, gender, cognitive level and several other factors (Ganguli et al., 2005; Brodaty et al., 2012; Koller et al., 2012). Accordingly, it is crucial to have data on mortality risk among dementia patients to determine the appropriate resources and services required for patients and their families (Garcia-Ptacek et al., 2014a). Numerous studies have investigated the risk of mortality in dementia patients although the availability of large cohorts or databases is rare. Most of the studies have focused on AD and VaD with little information about other dementia subtypes (Fitzpatrick et al., 2005). Furthermore, the factors that influence the risk of mortality might differ according to diagnosis (Clarke et al., 1995; Rountree et al., 2012). For these reasons, further studies that explore dementia mortality risk using large cohorts and registry databases are essential to obtain more valuable information on managing the disease (Lönnroos et al., 2013).

The establishment of SveDem offered a great opportunity to address the limitations of mortality studies by examining a very large cohort of dementia patients with a wide variety of dementia subtypes and with all their records available on the web (Garcia-Ptacek et al., 2014a; Table 2). A comparison of mortality risk among the most frequent dementia types in SveDem patients showed that VaD corresponds with the highest crude mortality rate while FTD is associated with the highest adjusted mortality risk. In contrast, the risk was lowest in AD patients. Higher age, male gender, institutionalization, lower cognition as scored by the MMSE test and a greater number of consumed medications at diagnosis were all factors that corresponded with higher risk of mortality (Garcia-Ptacek et al., 2014a). On the other hand, registry-based studies reported that body mass index (BMI) has a different role as the higher BMI the patient has at the time of dementia diagnosis, the lower risk of mortality the patient had. These results remained true for male patients up to (25–29.9 kg/m^2^) compared to females only up to (24.9 kg/m^2^) which might be explained by the social and biological differences between males and females (García-Ptacek et al., 2014b; Jang et al., 2015).

**TABLE 2:** Registry-based studies on dementia mortality risk.

#	Title	Year	Registry data used	Number of patients	Follow up duration	From	To	Purpose	Conclusion	References
1	No Significant Difference in Cognitive Decline and Mortality between Parkinson’s Disease Dementia and Dementia with Lewy Bodies: Naturalistic Longitudinal Data from the Swedish Dementia Registry	2018	SveDem	1110 DLB + 764 PDD			Dec 30th 2015	Compare cognitive decline and mortality between patients with Dementia with Lewy Bodies and Parkinson’s Disease Dementia	No significant difference in rate of cognitive decline and mortality between DLB and PDD	Fereshtehnejad et al., 2018
2	Mortality Risk after Dementia Diagnosis by Dementia Type and Underlying Factors: A Cohort of 15,209 Patients based on the Swedish Dementia Registry	2014	SveDem	15209				Analyze the risk of mortality in a large cohort of dementia patients	Male gender, consuming higher medications, age, and worse cognition, all were associated with increased death risk of dementia patients	Garcia-Ptacek et al., 2014a
3	Body-Mass Index and Mortality in Incident Dementia: A Cohort Study on 11,398 Patients from SveDem, the Swedish Dementia Registry	2014	SveDem	11398			Feb-13	Describe mortality risk relative to BMI	The risk of mortality in dementia patients decreased with the increase in BMI	García-Ptacek et al., 2014b
4	Body Mass Index and Mortality Rate in Korean Patients with Alzheimer’s Disease	2015	CREDOS	1911		Mar 2006	Dec 2010	Study the relationship between body mass index and mortality rate in AD patients	Patients with underweight had higher risk of mortality compared to normal weight AD patients	Jang et al., 2015
5	Causes of Death According to Death Certificates in Individuals with Dementia: A Cohort from the Swedish Dementia Registry	2016	SveDem	28609	741 days		Dec-12	Explore death causes that associates with different types of dementia	Dementia was less stated as the main death factor, Cardiovascular problems were the most frequent cause of death while respiratory disease was higher in LBD patients.	Garcia-Ptacek et al., 2016

Investigating the actual cause of death has revealed that dementia is usually underreported as the cause of death irrespective of the dementia subtype. AD patients, unlike other dementias, had lower all-cause risk of death. Cardiovascular diseases were the most common cause of death among dementia patients especially those with VaD, while respiratory diseases accounted for more death cases among patients with dementia with Lewy bodies (DLB) (Garcia-Ptacek et al., 2016). In addition, there was no significant difference between DLB and PDD in terms of mortality risk (Fereshtehnejad et al., 2018).

Thus, dementia registries have enabled us to study the causes of mortality among large cohorts of dementia patients to better understand and predict mortality risk (Table 2). Predicting the risk of death in each dementia group or subtype might help decrease the mortality rates among dementia patients through providing each group of patients with the most suitable management plan to treat the comorbidities and avoid the risk of death, as well as improving the quality of their lives.

## Impact of Registries on Disease Management

### Diagnostic Procedures and Risk Factors

The early and accurate diagnosis of dementia is essential for better disease monitoring and cost reduction in terms of care. However, in real life practice, the assessment of dementia and AD always occur after the symptoms have already developed and the disease has declared itself. Until now, there have been no widely used biomarkers that can facilitate dementia diagnosis and predict the disease before symptoms become apparent. Accordingly, exploring the factors that are associated with dementia in large cohorts of patients is crucial to identify the risk factors and understand mechanisms to modify these factors or diagnose the disease in early stages. Dementia registries have facilitated the research in the area of diagnostics and risk factors (Table 3). Research studies based on NACC registry data suggest that different modifiable factors such as sleep disturbance, depression and traumatic brain injury, as well as the non-modifiable factors including genetic biomarkers as apolipoprotein E (APOE) or abnormal aggregation of proteins like alpha synuclein and TDP-43 as potential risks of dementia and AD development (Burke et al., 2016; Bayram et al., 2019). PRODEM data analysis showed that quantitative EEG markers can also be considered as electro-psychological markers that relate to dementia and AD diseases although they remain research tools and are not used clinically (Fruehwirt et al., 2019). In addition, Schaffert et al. (2020) used NACC registry data to investigate six modifiable and non-modifiable risk factors as contributors to early dementia onset by examining different autopsy confirmed samples of AD, mixed AD and LBD, and pure LBD. APOE ε4 allele and male sex increased the risk of early onset by approximately 2 to 3 years in AD and mixed AD + LBD, while in pure LBD, depression and high education predicted a 5.5-year earlier onset of dementia (Schaffert et al., 2020).

**TABLE 3:** Registry-based studies on dementia diagnostics and risk factors.

#	Title	Year	Registry data used	Number of patients	Follow up duration	From	To	Purpose	Conclusion	References
1	Associations between Comorbid TDP-43, Lewy Body Pathology, and Neuropsychiatric Symptoms in Alzheimer’s Disease	2019	NACC	221		Sep-05	Sep-17	Examine the association between the comorbid pathologies in AD and FTLD or DLB-like neuropsychiatric symptoms	TDP-43 is associated with aberrant motor activity while Lewy bodies are associated with anxiety, irritability, sleep behavior and appetite problems	Bayram et al., 2019
2	Associations between Depression, Sleep Disturbance and Apolipoprotein E in the development of Alzheimer’s Disease: Dementia	2016	NACC	11453		Sep-05	Sep-14	Investigate the effect of sleep disturbance, depression, and the apolipoprotein E (APOE) genotype on increasing Alzheimer’s disease risk	Sleep disturbance, depression and APOE ε4 genotype increase the risk of AD	Burke et al., 2016
3	Associations of event-related brain potentials and Alzheimer’s disease severity: A longitudinal study	2019	PRODEM	63	18 months			Investigate the correlation between ERB components and AD severity	P300 and N200 latency significantly correlated with AD severity	Fruehwirt et al., 2019
4	Risk factors for earlier dementia onset in autopsy-confirmed Alzheimer’s disease, mixed Alzheimer’s with Lewy bodies, and pure Lewy body disease	2020	NACC	1221		Sep-05	Aug-16	Study the modifiable and non-modifiable risk factors for earlier dementia onset in autopsy-confirmed samples of AD, mixed AD LBD, and pure LBD	Male sex and APOE ε4 allele increase the risk of early dementia onset in AD patients while depression increases the early onset in AD, LBD and AD + LBD	Schaffert et al., 2020
5	Alzheimer’s disease first symptoms are age dependent: Evidence from the NACC dataset	2015	NACC	7815				Study the relationship between age and AD	Patients at younger ages were more likely to experience non-memory and behavioral symptoms	Barnes et al., 2015
6	Non-memory cognitive symptom development in Alzheimer’s disease	2020	NACC	9484		2005	2016	Study how age affects the development of non-memory cognitive symptoms in AD patients	Patients with younger onset AD are more likely to develop progressive non-memory symptoms	Blenkinsop et al., 2020
7	APOE is a correlate of phenotypic heterogeneity in Alzheimer’s disease in a national cohort	2020	NACC	42 from ADC/1418 + 2608 from NACC				Compare the proportion of APOE e4 genotype carriers in aphasic vs. amnestic variants of Alzheimer’s disease (AD)	The proportion of APOE ε4 genotype is higher in amnestic AD manifestations	Weintraub et al., 2020
8	The Effect of the *APOE*ε*2*ε*4* Genotype on the Development of Alzheimer’s Disease (AD) and Mild Cognitive Impairment (MCI) in Non-Latino Whites	2020	NACC	20176		2005	Aug-18	Study the association between APOE ε2ε4 and development of AD and MCI in non-Latino Whites	APOE ε2ε4 genotype is associated with increased risk of AD and MCI in non-Latino whites	Ren et al., 2020
9	Age-dependent effects of APOE ε4 in preclinical Alzheimer’s disease	2016	NACC	5381		2005	Nov 2014	Investigate the effects of APOE ε4 genotype on age of onset and progression of AD	APOE ε4 carrier status significantly influence progression of normal individuals into MCI or AD in all age groups with a peak at 70–75	Bonham et al., 2016
10	APOE ε4 allele status in Korean dementia patients with severe white matter hyperintensities.	2011	CREDOS	439				Investigate the APOE ε genotypes and clinical features of dementia patients with severe WMH	APOE ε4 allele is more prevalent in AD patients than SIVD patients	Hong et al., 2011
11	Association Between White Matter Hyperintensity Severity and Cognitive Impairment According to the Presence of the Apolipoprotein E (APOE) ε4 Allele in the Elderly: Retrospective Analysis of Data from the CREDOS Study	2012	CREDOS	5077		Nov-05	Dec-11	Examine the association between WMH severity and cognitive function	Severe WMHs are associated with frontal/executive dysfunction	Son et al., 2012
12	Prevalence of Traumatic Brain Injury in Early vs. Late-Onset Alzheimer’s Disease	2016	NACC	11572		Sep-05	May-14	Examine the relationship between TBI and EOAD	TBI is a risk factor for EOAD	Mendez et al., 2015
13	History of traumatic brain injury interferes with accurate diagnosis of Alzheimer’s dementia: a nation-wide case-control study	2020	NACC	5687			June 2019.	Evaluate the impact of TBI on the accuracy of clinically diagnosed AD	TBI controls AD diagnostic accuracy	Pradeep et al., 2020
14	Quantitative EEG in Alzheimer’s disease: Cognitive state, resting state and association with disease severity	2014	PRODEM	79				Evaluate the usefulness of using cognitive qEEG in diagnosis	qEEG alterations recorded during mnestic activity are valuable marker for disease severity	Garn et al., 2014b
15	Using static and dynamic canonical correlation coefficients as quantitative EEG markers for Alzheimer’s disease severity	2014	PRODEM	79				Analyze the static and dynamic correlation between EEG synchrony and EEG channel groups		Waser et al., 2014
16	Quantitative EEG markers relate to Alzheimer’s disease severity in the Prospective Dementia Registry Austria (PRODEM)	2015	PRODEM	118				Determine which QEEG marker or combination of markers can be used in assessing AD severity	Both cognitive and resting state should be used for QEEG assessment	Garn et al., 2015
17	Quantifying synchrony patterns in the EEG of Alzheimer’s patients with linear and non-linear connectivity markers	2015	PRODEM					investigate the synchrony in EEG markers and their association with AD severity	EEG synchrony may reflect cerebral compensatory mechanisms in the early stages of AD	Waser et al., 2016
18	Electroencephalographic complexity markers explain neuropsychological test scores in Alzheimer’s disease	2014	PRODEM	79				Investigate the correlation between AD severity and signal complexity measures auto-mutual information, Shannon entropy and Tsallis entropy	Auto-mutual information is well suited to characterize disease severity in mild to moderate AD	Garn et al., 2014a
19	Predicting rapid cognitive decline in Alzheimer’s disease patients using quantitative EEG markers and neuropsychological test scores	2016	PRODEM	68	1 year			Investigate the possibility of using QEEG markers and neuropsychological test scores to Predict Rapid Cognitive Decline (RCD) among AD patients		Reyes-Coronel et al., 2016
20	Quantitative EEG Markers of Entropy and Auto Mutual Information in Relation to MMSE Scores of Probable Alzheimer’s Disease Patients	2017	PRODEM	79				Investigate the association between non-linear qEEG markers and mild- to moderate severity of AD		Coronel et al., 2017

#### Genetic Biomarkers and Non-modifiable Risk Factors

Several studies have investigated the mechanism and associated risk of each genotype of APOE (Genin et al., 2011; Liu et al., 2013), but the major limiting factor of these studies was the small sample size that cannot detect or confirm the association of each genotype with other risk factors. There is a need to study larger populations for more powerful and informative analyses.

The NACC data has greatly contributed to improving the research studies investigating the presentation of AD symptoms among a United States population cohort, as well as identifying the potential genetic biomarkers that are associated with dementia and their relevance to the other risk factors of the disease (Table 3). It is increasingly recognized that AD dementia does not always begin with memory complaints, as less typical non-memory symptoms like language problems, visuospatial dysfunction, dyspraxia, and others can present as well (Jacobs et al., 1994; Koedam et al., 2010). A study of the NACC cohort reported that patients, especially those with younger onset, experience non-memory cognitive or behavioral symptoms first in contrast to older patients who usually experience memory decline at their first presenting symptoms (Barnes et al., 2015).

Examining the influence of APOE ε4 on the development of non-memory cognitive symptoms such as language, visuospatial function, judgment, and attention symptoms in the NACC has shown that it has little influence on the development of cognitive symptoms. Accordingly, the effect of APOE happens early in the disease before the occurrence of cognitive symptoms while other genes are responsible for developing the non-memory symptoms (Blenkinsop et al., 2020). Another study used NACC data, as well to compare the proportion of APOE ε4 genotype in patients with memory or amnestic symptoms vs. non-memory or aphasic symptoms while controlling sex and age at symptom of onset and confirming data with a group of autopsy samples (Weintraub et al., 2020). The proportion of APOE ε4 was higher in patients with amnestic symptoms than in the aphasic patients which may mean that APOE ε4 is a risk factor that selectively increases memory symptoms (Weintraub et al., 2020). In addition, the risk of the APOE ε2ε4 was unclear as it encompasses both the toxic ε4 and protective ε2 copies (Ren et al., 2020). The role of APOE ε2ε4 in developing AD dementia or mild cognitive impairment (MCI) was examined in non-Latino white patients to control for the influence of ethnic group. APOE ε2ε4 was associated with an increased risk of AD and MCI similar to APOE ε3ε4 and ε4ε4 genotypes (Ren et al., 2020). Studying the effect of APOE ε4 genotype and age of onset on the progression of AD, data from NACC indicated that carrying APOE ε4 genotype influences the progression to MCI or AD in all ages especially in the older groups (70–75). These results will help tailoring the therapeutic interventions, when available, based on the genetic data and age of patient (Bonham et al., 2016).

Moreover, CREDOS data was also very useful in studying APOE prevalence in Korean dementia patients. Hong et al. (2011) compared the frequency of APOE ε4 and the difference in clinical features between AD and SIVD patients, with and without severe white matter hyperintensities (WMH). APOE ε4 was more prevalent in AD than SIVD patients while the frequency was not affected by the presence or absence of WMH (Hong et al., 2011). However, severe WMH and APOE ε4 have an interactive effect on memory cognitive function (Son et al., 2012; Table 3). Thus, the studies on genetic factors associated with dementia, particularly APOE genotype, were facilitated by the availability of registries data. Further research on this area would be important to better understand the disease pathogenesis and determine the genetic risks which might help with early disease diagnosis.

#### Modifiable Risk Factors

There is an increasing emphasis on identifying modifiable risk factors of dementia in order to develop better strategies to prevent the disease before symptoms can develop. Researchers have investigated a number of modifiable risks that are associated with dementia by analyzing data available in dementia registries (Table 3).

History of traumatic brain injury (TBI) is, for instance, an important risk factor associated with dementia (Sivanandam and Thakur, 2012). Mendez et al. (2015) used the NACC database to investigate whether TBI is associated with early onset AD (EOAD) and found that the prevalence of TBI in EOAD patients was higher than LOAD which suggests that TBI may be a specific risk for early onset disease (Mendez et al., 2015). Another study used NACC data and reported that TBI can lead to AD misdiagnosis and reduce the accuracy of diagnosis through an increased risk of false positive results which can have serious implications for patient treatment (Pradeep et al., 2020).

Sleep disturbance and depression are considered as potential modifiable AD risk factors, as well (Marques et al., 2010; Pistacchi et al., 2014). NACC data investigation showed that sleep disturbance, depression as well as APOE genotype are associated with subsequent diagnosis of AD dementia (Burke et al., 2016). Overall, mental health disorders are among the strong predictors of AD diagnosis, so enhancing individuals’ mental health might decrease the risk of AD developing later on (Xu J. et al., 2020). Further exploration of registries data would help identifying more risk factors and how they increase the risk of dementia and hence, determining the most appropriate strategies to avoid them.

#### Electro Psychological Markers

Quantitative electroencephalogram (qEEG) is a low cost, non-invasive tool that has potential to differentiate patients with AD from normal controls (Dauwels et al., 2010; Drago et al., 2011). Investigating qEEG analysis and cognitive parameters among large cohorts of dementia patients is essential to evaluate whether qEEG can be used as a diagnostic marker for AD (Klimesch, 1999; Garn et al., 2014b).

Prospective Dementia Registry Austria was a valuable source of data that facilitated qEEG research. Garn et al. (2015) reported that face-name encoding with eyes open was better than resting state and strongely correlated to MMSE scores. Early to moderate stages of AD are associated with qEEG changes that can be determined using signal processing (Garn et al., 2014b). Dauwels et al., studied various synchrony measures for AD diagnosis with EEG data and were able to identify the two synchrony measures that can distinguish MCI patients from control patients (Dauwels et al., 2010). In addition, qEEG markers were shown to be closely related to AD severity especially in patients with MMSE scores between 15 and 26 (Garn et al., 2014a,^2015^; Waser et al., 2014, 2016; Coronel et al., 2017). Moreover, PRODEM data showed that qEEG can be used to predict rapid cognitive decline in AD patients (Reyes-Coronel et al., 2016). Taking altogether, qEEG markers have potential as diagnostic markers for dementia but further investigations of their accuracy are required. Using data from different disease registries to study qEEG markers and their relevance to AD diagnosis may help establish if this tool could be used as part of the standard diagnostic procedures for AD (Table 3).

Overall, the existing dementia registries represent a valuable tool for studying the known risk factors of the disease, as well as, identifying new risk factors and markers that may help improve the accuracy of diagnostic procedures of the disease (Table 3). Non-modifiable risk factors including the genetic attributes as well as the modifiable risk factors such as sleep disturbance and mental health status have been investigated by using large baseline patient data sets as well as the longitudinal follow-ups in some registries. Therefore, expanding the registry data to include more patient history is essential to allow the determination of more associated risk factors.

### Registries and Drug Treatments

Dementia patients usually receive multiple drug treatments prior to and post-diagnosis. Centrally active drugs that are frequently prescribed to dementia patients can be mainly divided into two groups: anti-dementia drugs and drugs that treat behavioral and psychological symptoms of dementia. The first group includes acetylcholinesterase inhibitors (AChEIs) that are used in mild to moderate cases and memantine, and an NMDA receptor antagonist that is used in moderate to severe cases. Antipsychotics, anxiolytics, hypnotics, antidepressants, and sedatives are all used to handle different dementia associated behavioral symptoms (Bond et al., 2012; Turró-Garriga et al., 2015). In addition, dementia patients usually have comorbidities such as hypertension, diabetes mellitus or dyslipidemia, among others. Thus, dementia patients are frequently prescribed many other medications besides dementia-specific drugs (Andersen et al., 2011). Studying the effect of these treatments on large cohorts of dementia patients is crucial to determine the best management strategy of the disease and avoid the adverse side effects of polypharmacy.

Numerous studies have analyzed the data available on SveDem registry to explore the impact of medication consumption on dementia patients, as well as to compare the use of drugs among different disease subtypes (Table 4). These studies have shown that anti-dementia drugs are frequently used among all patients. In patients with DLB, the use of antipsychotics was significant, which is a concern due to the potential for severe side effects (Johnell et al., 2013). Women were more likely to be treated with cholinesterase inhibitors (ChEIs) than men (Johnell et al., 2013). Additionally, patients with early onset (EOAD) received ChEIs more than patients with late age of onset (LOAD), although they were generally receiving fewer overall drugs (Eriksson, 2014; Fereshtehnejad et al., 2014b). Patients on ChEIs were less likely to receive antipsychotics and anxiolytics than those not taking ChEIs (Fereshtehnejad et al., 2014b). However, the cautious use of medications should be considered as some medications that are used for treating dementia symptoms or even for treating other co-morbidities are xerostomic and usually cause hyposalivation and dry mouth which can increase the risk of oral complications, tooth extraction or loss (Lexomboon et al., 2018).

**TABLE 4:** Registry-based studies on dementia treatment.

#	Title	Year	Registry data used	Number of patients	Follow up duration	From	To	Purpose	Conclusion	References
1	Pharmaceutical consumption and cost in patients with dementia: A longitudinal study by the Registry of Dementias of Girona (ReDeGi) in Catalonia (Spain)	2015	ReDeGi	869	2007–2008 followed for 3 years (longitudinal)	2007	2010	Assess the pharmaceutical consumption and costs in dementia patients		Turró-Garriga et al., 2015
2	Differences in Drug Therapy between Dementia Disorders in the Swedish Dementia Registry: A Nationwide Study of over 7,000 Patients	2013	SveDem	7570	2007–2010	2007	2010	Examine the differences between the use of anti-dementia drugs and antipsychotics in dementia patients	The use of antipsychotics was high in dementia patients which might be worrying	Johnell et al., 2013
3	Differences in Routine Clinical Practice between Early and Late Onset Alzheimer’s Disease: Data from the Swedish Dementia Registry (SveDem)	2014	SveDem	5052				Investigate the differences in demographics, diagnostic work-up and pharmacological treatment between EOAD and LOAD	EOAD patients went through extended diagnostic work-up and received fewer treatment medications	Eriksson, 2014
4	Anti-Dementia Drugs and Co-Medication Among Patients with Alzheimer’s Disease	2014	SveDem	5907				Examine the use of anti-dementia drugs in patients with mild AD	Patients taking ChEIs received less antipsychotics and anxiolytics than other patients	Fereshtehnejad et al., 2014b
5	The Effect of Xerostomic Medication on Oral Health in Persons with Dementia	2018	SveDem					Investigate the association between xerostomic medications use and tooth loss in dementia patients	The continuous use of xerostomic medications can increase the risk for tooth loss in dementia patients	Lexomboon et al., 2018
6	Consumption of Pharmaceuticals in Primary Non-Alzheimer’s Degenerative Dementias	2012	ReDeGi	235				Investigate the sociodemographic, clinical data and drug consumption in patients with different dementia subtypes		López-Pousa et al., 2012
7	Trends in the Prescription and Long-Term Utilization of Antidementia Drugs Among Patients with Alzheimer’s Disease in Spain: A Cohort Study Using the Registry of Dementias of Girona	2017	ReDeGi	2992	2007–2014	2007	2014	Describe the characteristics of AD patients prescribed with antidementia drugs and the long-term trends with consumption		Calvó-Perxas et al., 2017
8	Profile and variables related to antipsychotic consumption according to dementia subtypes	2012	ReDeGi	1894	2007–2009	2007	2009	Examine the prevalence of AP consumption in dementia patients	APs are extensively used to treat BPSD	Calvó-Perxas et al., 2012a
9	Psychotropic Drugs in Patients with Alzheimer’s Disease: A Longitudinal Study by the Registry of Dementias of Girona (ReDeGi) in Catalonia, Spain	2014	ReDeGi	698	2007–2008 followed for 3 years (longitudinal)	2007	2010	Explore the use of psychotropic drugs in AD patients and examine the associated demographic and clinical variables		Calvó-Perxas et al., 2014
43	Adherence to Clinical Practice Guidelines during Dementia Work-Up in a Real-World Setting: A Study from the Registry of Dementias of Girona	2017	ReDeGi	475	2007–2011 and 2012–2015	2007	2015	Evaluate the adherence to GCP among registry specialists		Turró-Garriga et al., 2017
10	Anticholinergic Burden and Risk of Stroke and Death in People with Different Types of Dementia	2018	SveDem	39107	2.31 years		Dec 31 2014	Study the total anticholinergic cognitive burden (ACB) and how it associates with risk of stroke and death in people with different dementia subtypes	The risk of stroke and death increase in dementia patients with the use of anticholinergic medicines	Tan et al., 2018a
11	Acetylcholinesterase inhibitors and risk of stroke and death in people with dementia	2018	SveDem	44288				Study the effect of using acetylcholinesterase inhibitor (AChEI) on the risk of ischemic stroke and death in people with dementia	The risk of ischemic stroke and death in dementia patients decrease with the use of AChEIs	Tan et al., 2018b
12	Antipsychotic Treatment Associated with Increased Mortality Risk in Patients with Dementia. A Registry-Based Observational Cohort Study	2019	SveDem	56048			Aug 28 2016	Assess the mortality causes in dementia patients treated with typical and atypical antipsychotic drugs (APDs)	The use of atypical and typical APDs is associated with increased risk of death in patients with dementia	Schwertner et al., 2019
13	Antidepressants and mortality risk in a dementia cohort: data from SveDem, the Swedish Dementia Registry	2016	SveDem	20050		May 1st-2007	Oct 31-2013	Investigate the association between the use of antidepressants and mortality risk in dementia patients	The use of antidepressants is associated with lower risk of mortality especially in Alzheimer’s disease patients	Enache et al., 2016
14	Measuring anticholinergic exposure in patients with dementia: A comparative study of nine anticholinergic risk scales	2018	ReDeGi	5323	2007–2014	2007	2014	Describe the prevalence of anticholinergic consumption		Turró-Garriga et al., 2018
15	Pain treatment and its cost in old people with dementia: a descriptive analysis from the Registry of Dementias of Girona (ReDeGi)	2012	ReDeGi	1186	2007–2008 (cross-sectional)	2007	2008	Describe the use and cost of analgesics in dementia patients		Calvó-Perxas et al., 2013
16	Cholinesterase inhibitors in patients with diabetes mellitus and dementia: an open-cohort study of ∼23 000 patients from the Swedish Dementia Registry	2020	SveDem	22620		2007	Dec 2015	To assess the causes of mortality in patients with DM and dementia who were prescribed ChEI, and to compare the mortality rate in patients with or without DM who used ChEI	ChEI treatment was associated with lower mortality rate in patients with DM or AD	Secnik et al., 2020a
17	Long-term Effects of Cholinesterase Inhibitors on Cognitive Decline and Mortality	2021	SveDem	31,054		2007	2017	Investigate the effect of using ChEIs on slowing cognitive decline and decreasing dementia or death risk in Alzheimer’s dementia.	ChEI treatment is associated with cognitive benefits as well as decreased death risk.	Xu et al., 2021

The ReDeGi registers data have also been used to investigate of the use of pharmaceutical medications in dementia. A study by López-Pousa et al. (2012) focused on studying patients with non-AD dementia (n-ADD) subtypes in terms of clinical data and patterns of prescribing. They reported that the use of anti-dementia medications including ChEIs was very frequent among patients with n-ADD although those pharmaceuticals are not effective for n-ADD according to the guidelines (López-Pousa et al., 2012; O’Brien et al., 2017). Age also played a role in ChEIs use as with advances in age, there was more likelihood that the patient would be on memantine and ChEIs rather than ChEIs alone (Calvó-Perxas et al., 2017). Additionally, atypical antipsychotics were extensively used in dementia patients to treat their behavioral and psychological symptoms despite the increased risk of adverse effects (Calvó-Perxas et al., 2012a,^2014^). Besides the risk of adverse effects, higher pharmaceutical use increases the costs of dementia disease management (Turró-Garriga et al., 2015). Accordingly, the risk-benefit ratio should be considered prior to prescribing treatments to dementia patients. Furthermore, guidelines should be improved to recommend the most effective treatment strategy for severe dementia patients (Turró-Garriga et al., 2017).

As mentioned earlier, the consumption of medication at the time of diagnosis is associated with higher risk of mortality (Garcia-Ptacek et al., 2014a,^2016^; García-Ptacek et al., 2014b). A wide range of medications are prescribed to dementia patients to treat behavioral symptoms, but they can be associated with serious side effects. Several studies assessed the effectiveness of the commonly used medications in dementia and their influence on the risk of mortality (Calvó-Perxas et al., 2013; Enache et al., 2016; Tan et al., 2018a,b; Schwertner et al., 2019). Although antidepressants are commonly used to treat dementia patients, they may neither increase nor decrease the risk of mortality. However, it was shown that the use of antidepressants during prodromal stages and prior to dementia diagnosis for 3 consecutive years is associated with decreased risk of mortality especially in AD patients (Enache et al., 2016).

Medications with anticholinergic properties are commonly used in dementia patients (Roe et al., 2002). Turró-Garriga et al., described the prevalence of anticholinergic exposure in dementia patients to be 36.3–69% determined based on the severity of the disease (Turró-Garriga et al., 2018). However, the aggregated effects of anticholinergic medicines, known as anticholinergic burden, have significant adverse effects on cognitive and physical functions of patients (Fox et al., 2014a; Gray et al., 2015). Tan et al., investigated the effect of anticholinergic burden on stroke outcomes and mortality risk in dementia patients. They reported that an increased risk of stroke is usually associated with the use of anticholinergic medicines and hence it increases the risk of mortality (Tan et al., 2018a). In contrast, the use of ChEIs in dementia and AD patients was associated with lower risk of stroke and death (Tan et al., 2018b). Moreover, the impact of antipsychotics use on mortality risk was also explored. The use of both typical and atypical antipsychotic drugs was associated with higher mortality risk in patients with dementia (Schwertner et al., 2019).

Disease registries have enabled clinicians and researchers to gain insights into the commonly used medications and their effectiveness and adverse effects in managing dementia. Using SveDem (Xu et al., 2021) found that cholinesterase inhibitors use was associated with higher memory over time (as measured by MMSE) and lower death compared to patients not using Cholinesterase inhibitors. Similarly, using SveDem (Secnik et al., 2020a), found that cholinesterase inhibitors use was associated with reduced mortality. One study found that cholinesterase inhibitors use reduces neurological and functional problems in pre-stroke dementia (Wakisaka et al., 2021). By using different registries, cholinesterase inhibitors were found to be associated with better cognition in patients with vascular dementia (Battle et al., 2021).

However, more data on the medications used pre- and post- dementia diagnosis should be considered when registering patients. The duration of treatment and the actual doses are also important to consider. Having an integrated patient registry that contains all the information about treatment history will help with studying the effectiveness of different anti-dementia medications and predicting the adverse effects that could arise when using multiple medications and thereby help improve the current treatment approaches and allow for better disease management. Identifying more precised treatment approaches will also lead to enhanced patients’ quality of life which is the most important objective of all registry initiatives as well, as the studies build on them.

## Dementia Registries Advance Our Knowledge of the Disease

### Association of Dementia With Other Diseases

To date, little attention has been paid to research on the specific healthcare needs of dementia patients. Dementia is more common in people with older ages who often have other medical problems or comorbidities (Fox et al., 2014b). Several comorbidities including diabetes, cardiac diseases, pulmonary diseases, musculoskeletal disorders, mental disorders and others have been reported commonly in dementia patients and shown to reduce their quality of life (Schubert et al., 2006; Martín-García et al., 2013). In addition, the elimination of some of these comorbidities including diabetes, depression and hypertension may help reduce the percentage of new cases diagnosed with dementia (Livingston et al., 2017). Accordingly, early prediction, diagnosis and management of new conditions that are associated with dementia patients may help decrease the severity of the disease and improving their quality of life (Marengoni et al., 2009; Thorpe et al., 2012). The availability of data recorded for patients with dementia in the several international registries has allowed researchers to investigate the risk of other diseases that may be associated with dementia and to create comorbidity profiles for the different dementia subtypes (Fereshtehnejad et al., 2014a; Table 5).

**TABLE 5:** Registry-based studies on dementia comorbidities.

#	Title	Year	Registry data used	Number of patients	Follow up duration	From	To	Purpose	Conclusion	References
1	Association of Hypercholesterolemia with Alzheimer’s Disease Pathology and Cerebral Amyloid Angiopathy	2020	NACC	3508 (autopsies)		2005	2017	Study the association between hypercholesterolemia (HC) and risk of AD neuropathology	HC significantly associated with increased severity of AD	Xu C. et al., 2020
2	Intensive control of hypertension and risk of Alzheimer’s dementia in older adults with depression	2020	NACC	5587		Sep 2005	Mar 2019	Investigate the association between intensive control of systolic blood pressure and the risk of AD dementia	Controlling systolic BP intensively in older adults with hypertension and depression was associated with increased risk of AD	Yeung et al., 2020
3	Diabetes in a Large Dementia Cohort: Clinical Characteristics and Treatment from the Swedish Dementia Registry	2017	SveDem	29630 (4881 with diabetes)		May 2007	Dec 2012	Study the clinical characteristics and pharmacological treatment in dementia patients with diabetes	Diabetes was associated with younger age at the time of dementia diagnosis, as well as less administered dementia medications which might suggest less dementia control	Secnik et al., 2017
4	Interaction between APOE genotype and diabetes in cognitive decline	2020	NACC	24967		Sep 2005	Sep 2018	Investigate the association between diabetes and cognitive decline and neuropathological changes depending on APOE genotype	Diabetic patients showed accelerated cognitive decline especially those who are APO2 or APOE3 carriers while APOE4 carriers were already vulnerable to vascular impairment	Shinohara et al., 2020
5	Heart failure and dementia: survival in relation to types of heart failure and different dementia disorders	2015	SveDem and RiksSvikt	775		2007	Oct 2013	Investigate the association between dementia and its subtypes and heart failure (HF) with an assessment to the survival of dementia patients	Vascular dementia was the most common dementia disorder, neither HF nor dementia were associated with survival	Cermakova et al., 2015
6	Management of Acute Myocardial Infarction in Patients with Dementia: Data from SveDem, the Swedish Dementia Registry	2017	SveDem	525	228 days	May 2007	Dec 2012	Investigate the factors regulating the use of invasive procedures in managing acute myocardial infarction (AMI) in dementia patients and whether these procedures will enhance their survival	Age and cognitive status are factors that determine using invasive procedures in the management of AMI patients with better survival	Cermakova et al., 2017
7	Acute Stroke Care in Dementia: A Cohort Study from the Swedish Dementia and Stroke Registries	2018	SveDem and Riksstroke	1356 Dementia + AIS/6755 AIS + no dementia		2010	2014	Evaluate the hospital management of acute ischemic stroke (AIS) with and without dementia	Ischemic stroke patients with dementia had equal access to stroke unit care as non-dementia patients, their stays were shorter, and they received less diagnostic assessments	Zupanic et al., 2018
8	Secondary Stroke Prevention After Ischemic Stroke in Patients with Alzheimer’s Disease and Other Dementia Disorders	2020	SveDem and Riksstroke	1410 dementia with IS (332 AD)/7150 non-dementia IS	3 years	2007	2014	Compare secondary stroke prevention rate after 3 years of first ischemic stroke in dementia and non-dementia patients	Dementia is a predictor of the treatment plan used post-stroke for secondary stroke prevention	Zupanic et al., 2020
9	Prestroke Mobility and Dementia as Predictors of Stroke Outcomes in Patients Over 65 Years of Age: A Cohort Study from The Swedish Dementia and Stroke Registries	2018	SveDem and Riksstroke	1689 Dementia + stroke/7973 non-dementia + stroke	3 months	2007	2014	Investigate the prestroke mobility dependency and dementia and their association with functioning and mortality outcomes	A high burden after stroke was found in dementia patients	Garcia-Ptacek et al., 2018
10	Stroke as a Cause of Death in Death Certificates of Patients with Dementia: A Cohort Study from the Swedish Dementia Registry	2019	SveDem	49823		May 2007	Dec 2014	Describe the characteristics of dementia patients dying from stroke	Stroke was underestimated as a cause of death in death certificates of dementia patients	Subic et al., 2018b
11	Treatment of Atrial Fibrillation in Patients with Dementia: A Cohort Study from the Swedish Dementia Registry	2018	SveDem	8096		2007	2014	Study the benefits and complications of using anticoagulants in the treatment of atrial fibrillation in patients with dementia	Warfarin treatment is associated with lower risk of developing stroke and death which indicates its benefits in treating AF in patients with dementia	Subic et al., 2018a
12	Thrombolysis in acute ischemic stroke in patients with dementia	2017	SveDem and Riksstroke	1356 Dementia + AIS/6755 non-dementia + AIS	3 months	2010	2014	Assess the outcomes of using intravenous thrombolysis for treating AIS in dementia patients	There were no significant differences among dementia or non-dementia patients receiving IVT	Zupanic et al., 2017
13	Increased risk of epilepsy in patients registered in the Swedish Dementia Registry	2019	SveDem	81192 Dementia/223933 Control			Dec 2017	Investigate the incidence and risk factors of epilepsy in dementia patients	The risk of epilepsy increases in patients with dementia particularly young-onset Alzheimer’s disease	Zelano et al., 2020
14	Dementia Diagnosis Is Associated with Changes in Antidiabetic Drug Prescription: An Open-Cohort Study of ∼130,000 Swedish Subjects over 14 Years	2020	SveDem	133318 (12284 with dementia and DM)		2005	2018	Review the pharmacological adaptation in diabetes treatment in dementia patients	Dementia patients are less likely to receive new diabetes medications and are more likely to have insulin dispensation	Secnik et al., 2020b
15	Cholinesterase inhibitors in patients with diabetes mellitus and dementia: an open-cohort study of ∼23 000 patients from the Swedish Dementia Registry	2020	SveDem	22660 (3176 with DM)		2007	Dec 2015	Evaluate the effectiveness of ChEIs in patients with DM and dementia	ChEIs showed to reduce mortality risk in patients with DM and AD or dementia, particularly donepezil and galantamine	Secnik et al., 2020a
16	Risk of epilepsy diagnosis after a first unprovoked seizure in dementia	2020	SveDem	1039/743 controls		2007	2017	Study the risk of epilepsy diagnosis in patients with dementia after an unprovoked seizure	Epilepsy cannot be diagnosed after having a first seizure on the basis that patients have dementia	Mahamud et al., 2020

The relationship between hypercholesterolemia (HC) and AD was among the comorbidities that warranted investigation as HC was shown to accelerate AD pathology in animal studies (Xu C. et al., 2020). Using neuropathological and clinical data of the large national sample with autopsies from NACC, a group of researchers were able to investigate the association of hypercholesterolemia (HC) with AD. They suggested that the severity of AD disease was increased with the existence of HC. The association of the two diseases was significant even when the model was adjusted to control the ApoE genotype (Xu C. et al., 2020). In addition, data from NACC helped to investigate the association between controlling systolic blood pressure and the risk of AD. Survival analyses in older adults with treated hypertension and comorbid depression showed that the intensive control of systolic blood pressure was associated with risk of AD. The actual reasons are not yet fully understood, however, the cautious management of hypertension in these vulnerable groups will be needed (Yeung et al., 2020).

Diabetes Mellitus was also one of the comorbidities that needed investigation as several studies suggested its association with the risk of dementia and AD (Lu et al., 2009; Mittal and Katare, 2016). Data from SveDem was used in a registry-based study to investigate large cohort of dementia patients with diabetes and its impact on their clinical characteristics and treatment. The study reported that diabetic patients were younger than control patients when diagnosed with dementia and they received less dementia medications (Secnik et al., 2017). In addition, researchers initiated an open-cohort study to evaluate the effectiveness of some dementia medications including ChEIs in patients with diabetes. They reported that patients with diabetes and dementia or AD had reduced mortality risk when receiving ChEIs, particularly donepezil and galantamine (Secnik et al., 2020a). On the other hand, Scenik et al., examined the pharmacological changes in the treatment of diabetes in patients with dementia. They indicated that dementia patients were less likely to receive new diabetes medications and they had higher insulin dispensation ratio than non-dementia controls (Secnik et al., 2020b). Reviewing NACC data showed also that diabetes is associated with accelerated cognitive decline in patients with APOE2 and APOE3 genotypes but not APOE4 carriers (Shinohara et al., 2020). Thus, dementia registries data have contributed to our understanding of the relation between dementia and diabetes and the management of patients with both comorbidities. Further research will be crucial to determine the most appropriate treatment plan to increase patients’ survival and decrease the severity of cognitive impairment (Secnik et al., 2017). Understanding the mechanisms by which diabetes is associated with dementia will help, as well as understanding new management approaches.

Cognitive impairment frequently occurs with cardiovascular diseases (CVD) and heart failure (HF) but the underlying mechanisms remain unclear. Various studies reported that cognitive impairment is a risk factor of CVD which make CVD and HF patients are at increased risk of developing dementia or AD (Angermann et al., 2012; Roher, 2015). To investigate the relationship between HF and dementia, researchers used SveDem data to study the difference in prognosis in patients with HF and dementia, assess the proportion of different dementia subtypes among HF patients and describe their clinical characteristics. They found that vascular dementia was the most common subtype associated with HF with no associated survival in either dementia or HF patients (Cermakova et al., 2015). SveDem data was also used to study patients with acute myocardial infarction (AMI) and dementia, and whether using invasive procedures in their management would affect their survival. Using invasive procedures can enhance the survival of patients with dementia and AMI (Cermakova et al., 2017). Moreover, Subic et al., studied the benefits and complications associated with anticoagulant therapy in patients with dementia and atrial fibrillation (AF). Patients on warfarin had decreased risk of developing ischemic stroke unlike those on antiplatelets which increased Ischemic Stroke risk. Using warfarin was associated with lower mortality rate in dementia patients which indicates the benefits of warfarin on stroke prevention (Subic et al., 2018a).

Cerebrovascular disease usually coexists with dementia and dementia patients are at increased risk of developing strokes which increases their disability and dependency (Hay et al., 2017; Subic et al., 2017). Accordingly, managing dementia patients become more challenging when acute stroke exists (Saposnik et al., 2011). Previous studies proposed that dementia negatively affects survival and outcomes of stroke, but little attention was given to the management of patients with dementia and stroke (Saposnik et al., 2011, 2012). Data from SveDem registry allowed researchers to investigate the association between dementia and stroke, and the provided care plans (Table 5). Gracia-Ptacek et al., investigated prestroke mobility dependency and its association with dementia and post-stroke outcomes. They demonstrated that dementia patients were more likely to have impaired functionality and dependency prior to stroke. After stroke, dementia patients had a higher likelihood of developing poor mobility, disability and accordingly increased residential assistance and mortality (Garcia-Ptacek et al., 2018). Investigating this higher mortality rate in dementia patients revealed that most of the dementia patients who had ischemic stroke in their death certificates were not registered in the Swedish stroke registry (Riksstroke), which makes the accuracy of these death certificates questionable (Subic et al., 2018b). Zupanic et al., studied the hospital management of acute ischemic stroke patients with dementia. They reported that there was no difference in most of the aspects of stroke care between patients with or without dementia as all had equal access to stroke unit care (Zupanic et al., 2018). SveDem data was used, as well, to study the treatment plans of secondary stroke prevention to decrease cognitive decline by comparing between patients with and without dementia. The study reported that patients with dementia were more likely to receive antiplatelets post-stroke while they were less likely to receive anticoagulants, statins and blood pressure lowering medications (Zupanic et al., 2020). In addition, the administration of intravenous thrombolysis showed no significant difference among patients with or without dementia (Zupanic et al., 2017). These results may represent a new target to improve the quality of care of dementia patients.

Epilepsy has also been examined to investigate whether its risk increases with dementia. Examining SveDem large cohorts, Zelano et al., found that the risk of subsequent epilepsy increases with dementia (Zelano et al., 2020). When studying the risk of epilepsy diagnosis after the first unprovoked seizure in patients with dementia, researchers suggested that epilepsy cannot be diagnosed after the first seizure only on the basis that a patient has dementia (Mahamud et al., 2020). Rather, researchers should investigate how to predict the risk of epilepsy in dementia patients (Mahamud et al., 2020). More studies are needed to understand the exact mechanisms for the association between epilepsy and dementia in order to prevent the occurrence of epilepsy in dementia patients and enhance its management.

In conclusion, the data available in different dementia registries helped us advancing our knowledge of the disease and how it associated with different medical comorbidities. Investigating the clinical characteristics of large cohorts of patients will allow the determination of the most appropriate ways to manage dementia patients with comorbidities, with the ultimate goal of improving the quality of life of dementia patients.

## Conclusion

Dementia registries have significantly improved our knowledge of dementia and have contributed to enhancing dementia research. The availability of large data sets has greatly impacted on clinical practice and will help redirect government policies as well as the healthcare services to better reflect patient needs, with clinical and economical benefit. The growth in registries data has enabled researchers to study different cohorts to determine the risk factors of the disease, prevalence and identify new disease markers for earlier diagnosis and better management. In addition, an improved understanding of the socio-demographic and socioeconomic factors that can influence patient outcome will help lead to more precision and targeted patient centered treatment.

Many national and international dementia registries have improved our knowledge of the disease through the use of observational, cross-sectional and longitudinal data. SveDem and ReDeGi, for instance, significantly contributed to our knowledge on the disease prevalence and differences in patients’ characteristics which will be important to develop new algorithms to predict the disease. In addition, greater knowledge of mortality risks will help provide patients with the appropriate services that can improve their survival. NACC, PRODEM, and CREDOS also contributed information regarding the modifiable and non-modifiable risk factors that are associated with dementia. Understanding the underlying mechanisms of how these risk factors contribute to the pathogenesis of dementia will be crucial to early diagnosis and new treatments for the disease.

Moreover, investigating patients’ outcomes with different treatment strategies for dementia patients with or without comorbidities will help determine the most appropriate management strategy for each case to improve the quality of their lives.

Given the benefits of dementia registries and their contribution to improved understanding of risk and outcomes, the development of dementia registries should be considered in all countries where such gaps exist. Harmonization efforts of dementia registries across countries should also be explored to determine whether risk and protective factors are the same in different countries and continents and to share learnings in how best to tackle the global challenge of dementia.

## Author Contributions

SH contributed to conceptualization and writing-original draft. MS, AM, and BL contributed to supervision and writing-review and editing. YR contributed to writing-review and editing. All authors contributed to the article and approved the submitted version.

## Conflict of Interest

The authors declare that the research was conducted in the absence of any commercial or financial relationships that could be construed as a potential conflict of interest.

## Publisher’s Note

All claims expressed in this article are solely those of the authors and do not necessarily represent those of their affiliated organizations, or those of the publisher, the editors and the reviewers. Any product that may be evaluated in this article, or claim that may be made by its manufacturer, is not guaranteed or endorsed by the publisher.

## Abbreviations

AD: Alzheimer’s disease
ADD: Alzheimer’s disease dementia
n-ADD: non-Alzheimer’s disease dementia
VaD: vascular dementia
FTD: frontotemporal dementia
DLB: dementia with Lewy bodies
PDD: Parkinson’s disease dementia
EOD: early onset dementia
LOD: late-onset dementia
LBD: Lewy body disease
MCI: mild cognitive impairment
CERAD: consortium to establish a registry for Alzheimer’s disease
ReDeGi: Registry of Dementias of Girona
SveDem: Swedish Dementia Registry
NACC: National Alzheimer’s Coordinating Center
CREDOS: Clinical Research Center for Dementia of South Korea
PRODEM: Prospective Dementia Registry Austria
MMSE: Mini-Mental State Examination
BMI: body mass index
EEG: electroencephalogram
qEEG: quantitative electroencephalogram
AChEIs: acetylcholinesterase inhibitors
APOE: apolipoprotein
SIVD: Subcortical Ischemic Vascular Dementia
WMH: white matter hyperintensities
CVD: cardiovascular diseases
HF: heart failure
AMI: acute myocardial infarction
AF: atrial fibrillation
Riksstroke: Swedish stroke registry.
